# Initial Assessment and Monitoring of Patients with Chronic Hypoparathyroidism: A Systematic Current Practice Survey

**DOI:** 10.1002/jbmr.4698

**Published:** 2022-11-14

**Authors:** Stan Van Uum, Muhammad Shrayyef, Iman M’Hiri, Karel Dandurand, Dalal S. Ali, John P. Bilezikian, Michael T. Collins, Michael Mannstadt, Mishaela R. Rubin, Heide Siggelkow, Gaia Tabacco, Yu-Kwang Donovan Tay, Tamara Vokes, Karen K. Winer, Liang Yao, Gordon Guyatt, Lars Rejnmark, Aliya A. Khan

**Affiliations:** 1 Department of Medicine, Western University, London, ON, Canada; 2 Dept of Medicine, University of Toronto, Toronto, ON, Canada; 3 Bone Research and Education Centre, Oakville, ON, Canada; 4 Division of Endocrinology and Metabolism, McMaster University, Hamilton, ON, Canada; 5 Vagelos College of Physicians and Surgeons, Columbia University, New York, NY, USA; 6 National Institute of Dental and Craniofacial Research, National Institutes of Health, Bethesda, MD, USA; 7 Endocrine Unit, Massachusetts General Hospital and Harvard Medical School, Boston, MA, USA; 8 Clinic of Gastroenterology, Gastrointestinal Oncology and Endocrinology, University Medical Center Goettingen, Goettingen, Germany; 9 MVZ Endokrinologikum Goettingen, Goettingen, Germany; 10 Unit of Endocrinology and Diabetes, Campus Bio-Medico University of Rome, Rome, Italy; 11 Department of Medicine, Sengkang General Hospital, Singhealth and Duke-NUS Medical School, Singapore, Singapore; 12 University of Chicago, Chicago, IL, USA; 13 Eunice Kennedy Shriver National Institute of Child Health and Human Development, National Institutes of Health, Bethesda, MD, USA; 14 Department of Health Research Methods, Evidence and Impact, McMaster University, Hamilton, ON, Canada; 15 Dept of Endocrinology and Internal Medicine, Aarhus University hospital, Aarhus, Denmark

**Keywords:** HYPOPARATHYROIDISM, COMPLICATIONS, EXPERT, MONITORING, SURVEY

## Abstract

Chronic hypoparathyroidism (HypoPT) is associated with significant morbidity and impaired quality of life (QoL). The goals of management for chronic HypoPT include improvement in QoL and the prevention of both hypo- and hypercalcemia symptoms and long-term complications. Several groups have provided consensus statements and guidelines on the management of HypoPT; however, due to limited evidence, these recommendations have largely been based on literature reviews, expert opinion, and consensus statements. The objective of this study was to use a systematic approach to describe current practice for the initial assessment and follow-up of patients with chronic HypoPT. We developed a survey asking experts in the field to select the responses that best reflect their current practice. The survey found no differences in responses between nonsurgical and postsurgical patient assessment. For new patients, respondents usually performed an assessment of serum lab profile (calcium [either albumin-adjusted or ionized], magnesium, creatinine, phosphate, 25-hydroxyvitamin D), 24-hour urine (creatinine, calcium), and a renal ultrasound to evaluate for the presence of nephrocalcinosis or nephrolithiasis. For follow-up patients, most respondents perform blood tests and urine tests every 6 months or less frequently. The reported clinical practice patterns for monitoring for complications of chronic HypoPT vary considerably among respondents. Based on the responses in this systematic expert practice survey, we provide practice suggestions for initial assessment and follow-up of patients with chronic HypoPT. In addition, we highlight areas with significant variation in practice and identify important areas for future research. © 2022 The Authors. Journal of Bone and Mineral Research published by Wiley Periodicals LLC on behalf of American Society for Bone and Mineral Research (ASBMR).

## Introduction

Hypoparathyroidism (HypoPT) is a rare endocrine disorder characterized by hypocalcemia and hyperphosphatemia in the presence of inappropriately normal, low, or undetectable levels of parathyroid hormone (PTH).^(1)^ HypoPT is associated with significant morbidity and impaired quality of life (QoL). Hypocalcemia may cause classical symptoms including numbness and tingling in the face, hands or feet, muscle cramps, depression, confusion, seizures, and laryngospasm. In addition, complications of chronic HypoPT may include basal ganglia calcifications, nephrocalcinosis, kidney stones, renal failure, and changes in bone mineral density (BMD).^(2,3)^ The bone mass in both cortical and cancellous compartments in HypoPT is increased as noted on dual-energy X-ray absorptiometry (DXA) and high-resolution peripheral quantitative computed tomography (HR-pQCT).^(4,5)^ Bone biopsies from the iliac crest in patients with HypoPT have shown increases in cortical thickness and cancellous bone volume due to increased trabecular thickness.^(6)^ However, the clinical effects of increased bone density and cortical thickness, as well as cancellous bone volume on fracture risk in patients with chronic HypoPT, are not known.^(7)^ There are currently no prospective data on fracture risk in individuals with HypoPT. Case control studies show no difference in fracture rates in comparison to the general population.^(8,9)^

The goals of chronic HypoPT management are the improvement of QoL and the prevention of hypo- and hypercalcemia symptoms and complications associated with abnormal mineral homeostasis. Several groups have provided consensus statements and guidelines on the management of HypoPT.^(2,3,10)^ These recommendations have been based on literature reviews, expert opinion, and consensus statements. In 2015, Bollerslev et al.^(10)^ and in 2019 Khan et al.^(3)^ concluded that data on the risk of complications had just started to emerge, and there are only a few clinical trials on treatment optimization, limiting the possibility of formulating detailed recommendations.^(11)^

We conducted a systematic review of monitoring strategies of chronic HypoPT. Only two studies were identified, neither of which compared different monitoring strategies in patients with chronic HypoPT.^(12,13)^ One study suggested monitoring serum parathyroid hormone levels every 6 or 12 months for up to 3 years in patients with chronic postoperative HypoPT. The other study suggested monitoring serum calcium in pregnant women with idiopathic HypoPT every 3 weeks over a 6-month period. This systematic review provides very limited evidence on how to monitor chronic HypoPT in clinical practice.

To begin addressing this gap in knowledge, we developed a survey to document current expert practice on initial evaluation and monitoring of patients with HypoPT. The objective of this survey was to use a systematic approach to describe the current practice of monitoring chronic HypoPT by the expert panel assembled to develop the parathyroid guidelines, both for hypoparathyroidism and primary hyperparathyroidism,^(14)^ and to derive evidence-based guidance for monitoring patients with chronic HypoPT. Task force members were selected based on their expertise and publications in the field, with consideration given to international geographic representation. The steering committee gave equal preference to men and women. We invited experts in endocrinology, endocrine surgery, and nephrology, as well as general endocrinologists, thereby enabling the development of multidisciplinary practical evidence-based guidelines for both adults and children. A critical aspect of this approach was that the survey asked respondents to choose responses that were closest to their actual current practice rather than base their responses on recommendations from published guidelines or consensus statements.

## Methods

This survey was developed by the HypoPT Evaluation and Management Working group of the International HypoPT Task Force, a global group of experts in this field.^(14)^ The objective was to describe practice patterns for monitoring of chronic HypoPT. The survey platform utilized was Qualtrics (https://www.qualtrics.com/), a web-based software used to administer and distribute surveys and generate reports. Qualtrics is a leading survey software and was selected based on its professional interface, security, and ability to generate response reports. Email invitations with the link to the survey were sent to all members of the International Parathyroid Task Force in December 2020, with reminder emails sent during each of the two following months. The survey was closed for submission on March 5, 2021. All information was collected anonymously and confidentially, with results being reported in aggregate format. We included all submitted responses in the study.

For the demographics of the respondents, we obtained information on practice setting, specialty, types of patients being monitored, number of years in practice, number of patients with HypoPT followed in active practice, and location of practice. The respondents were given the option to opt out for questions about patient groups not being managed in their practice.

### Assessment of new patients with chronic HypoPT

In this part of the survey, we asked respondents about their current assessment practice for patients with a new diagnosis of chronic HypoPT. This was done separately for two groups of patients, nonsurgical patients and postsurgical (confirmed HypoPT for at least 6 months following surgery) patients with chronic HypoPT. Importantly, we asked respondents to select the responses that were *closest to their current practice*. For each response option in this section, the respondents used a sliding tool (graded from 0% to 100%) to indicate the percentage of new patients in which they would perform the specified assessment. This was done for clinical assessment as well as for serum and urine laboratory tests. In addition, we inquired how frequently respondents would perform specified assessments for complications of chronic HypoPT. These included renal imaging studies, brain imaging for cerebral calcifications, ophthalmology examinations, BMD assessment, and QoL questionnaires. The detailed questions are listed in the supplement (Appendix S1).

### Follow-up of patients with chronic HypoPT

We asked respondents to provide information on their current follow-up practice for patients with chronic HypoPT, including nonsurgical patients and postsurgical patients. For both these patient groups, respondents were asked to respond separately for patients with stable disease and unstable disease based on respondents’ clinical judgment. This was done for clinical assessments as well as for serum and urine laboratory tests. We also inquired how frequently respondents would perform assessments for complications of chronic HypoPT and how often a QoL questionnaire would be completed. Using summation boxes for each response option, the respondents chose the percentages of patients for which they would perform the specified assessment, ranging from 0% to 100% with discrete 20% interval options. Respondents were instructed to choose percentages and create a response distribution, for example, for calcium monitoring they might indicate to monitor 20% of their patients every 3 months, 60% every 6 months, and 20% once yearly. We asked the respondents to ensure that the total of these responses would add up to 100%. The platform did not allow us to enforce this, but none of the response additions exceeded 100%. The detailed questions are listed in the supplement (Appendix S1).

For all responses, we evaluated how frequently a certain parameter was assessed and by what percentage of participants. For both the number of respondents and the number of patients assessed by individual respondents, we selected a minimum of 70% as the cut-off to demonstrate sufficient practice agreement between respondents to provide guidance for monitoring practice. For example, if testing for parameter X was done in 100% of patients by 65% of respondents and in 80% of patients by 10% of respondents, we would suggest monitoring for this parameter. In line with the guidelines developed by the Grades of Recommendation, Assessment, Development, and Evaluation (GRADE) working group,^(11)^ these are weak recommendations based on limited evidence, resulting in suggestions for clinical practice. Given that HypoPT is a rare disease with only a small number of affected patients and, hence, limited evidence, we elected not to make any suggestions *against* any aspect of monitoring practice. Once all responses were analyzed, they were discussed within our group, and, if required, additional guidance was provided.

## Results

The invitation for the survey was sent to 97 members of the International Task Force, and 70 members submitted a response, resulting in a 72% response rate. The demographics of the respondents are shown in Table 1. The majority of the respondents practiced in North America and Europe and had been in practice for over 25 years. The majority had between 10 and 100 patients with HypoPT in their practice. As the number of responses to the questionnaires about HypoPT management during pregnancy was very low, we decided not to include these responses in our analysis.

### Assessment of new patients

#### A: Chronic nonsurgical HypoPT

**Fig. 1.**
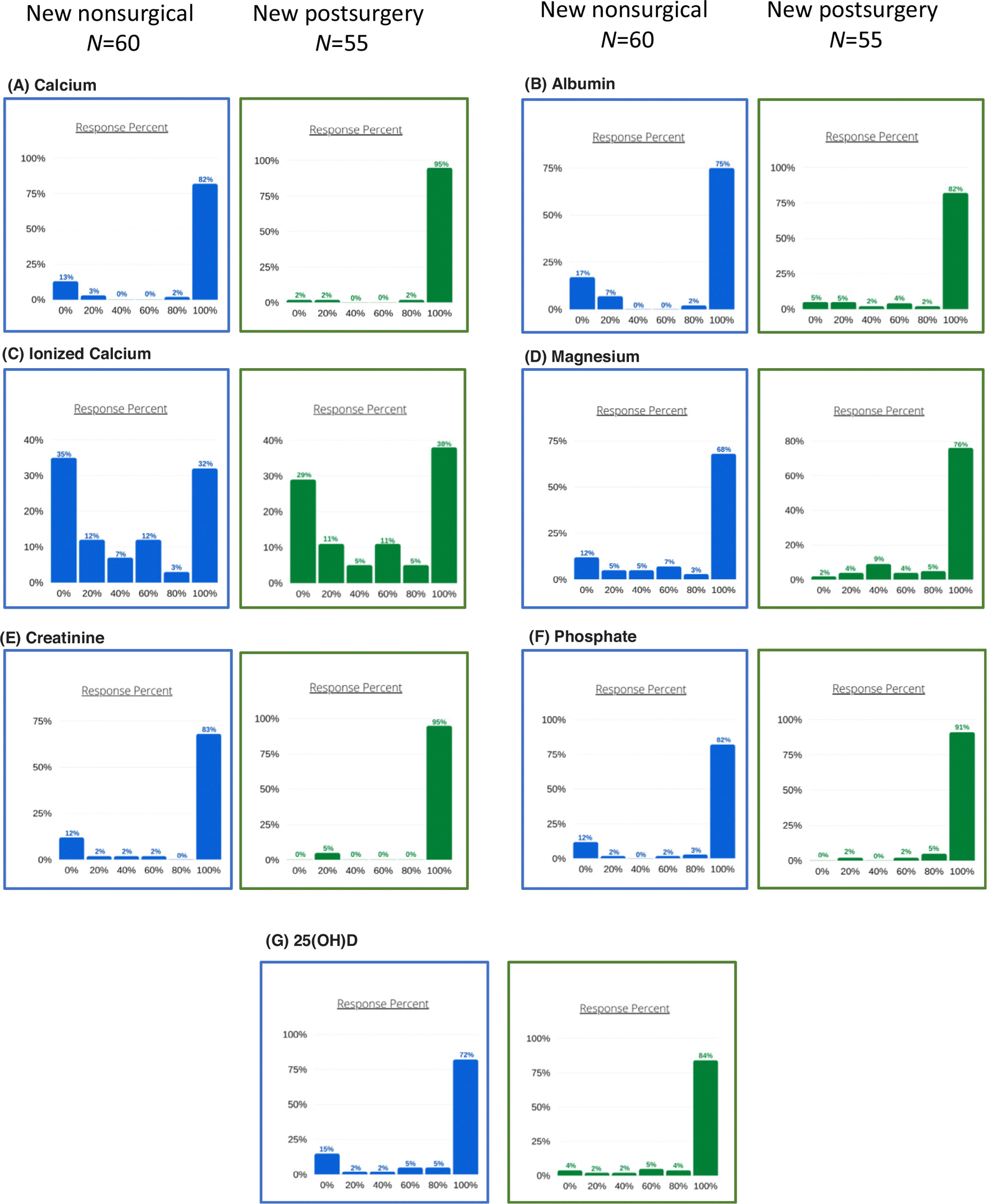
Reported assessment frequency for assessment of serum measurements in new HypoPT patients. The *x*-axis indicates the percentage of participants on whom assessment of the parameter was performed; the *y*-axis indicates the percentage of respondents who selected this option.

Sixty-five participants responded to this section. As part of their clinical assessment practice, 43% of respondents perform an assessment for the presence of anxiety and depression in 100% of patients, and 13% do so in 80% of patients. The results of biochemical monitoring practice when assessing new patients with chronic nonsurgical HypoPT are shown in Figure 1 (left panels). Respondents perform an assessment of serum calcium (either albumin-adjusted or ionized), magnesium, creatinine, phosphate, and 25-hydroxyvitamin D (25(OH)D).

**Fig. 2.**
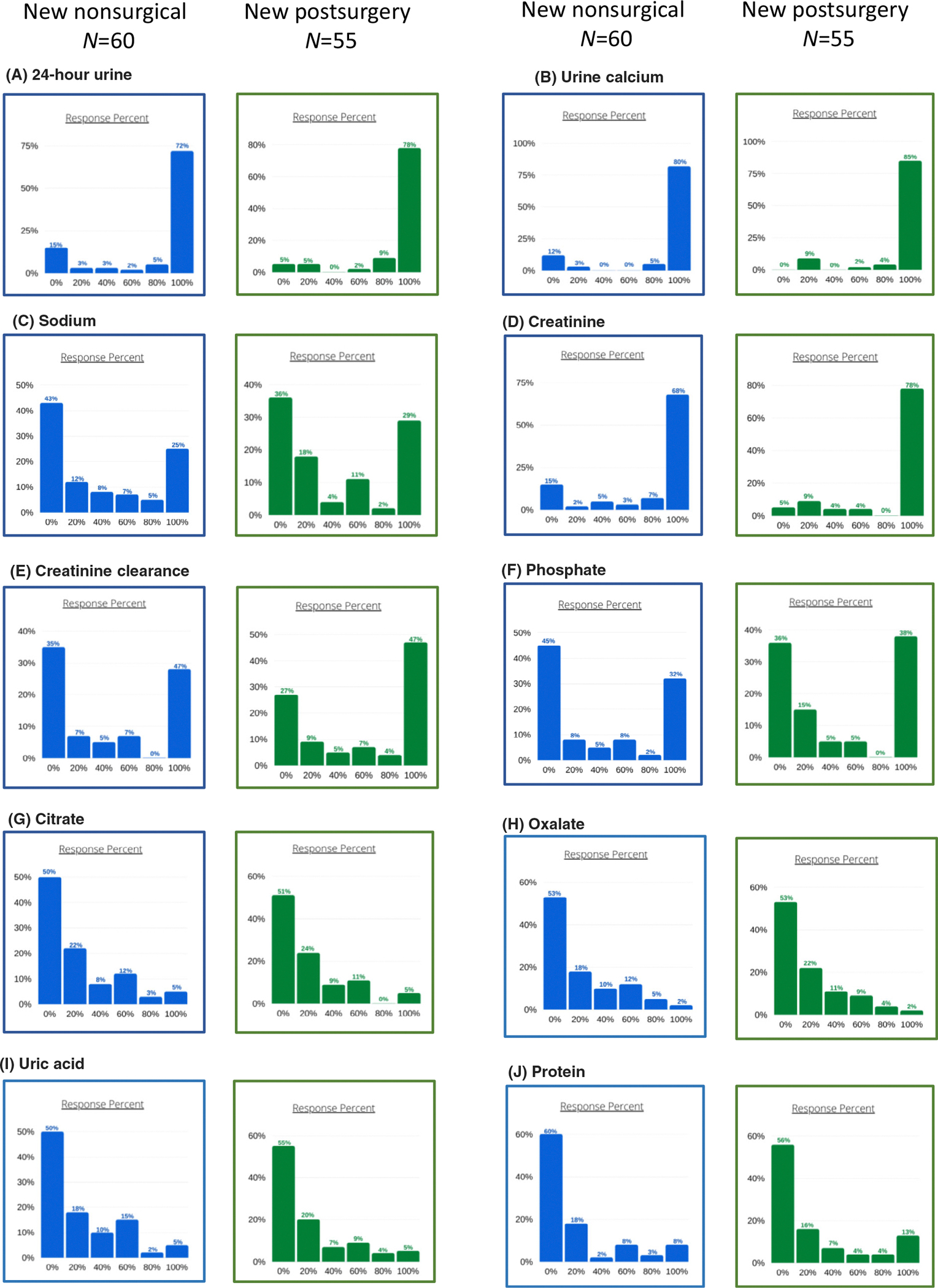
Reported assessment frequency for assessment of urine measurements in new HypoPT patients. The *x*-axis indicates the percentage of participants on whom assessment of the parameter was performed; the *y*-axis indicates the percentage of respondents who selected this option.

The results for 24-hour urine laboratory testing practice when assessing new patients with chronic HypoPT are shown in Figure 2 (left panels). Respondents usually measure 24-hour urinary creatinine and calcium. Measurements of other parameters, including metabolic parameters for kidney stone formation risk (citrate, oxalate, uric acid), are performed less frequently in most patients. Spot urine tests for calcium and creatinine excretion were performed by 15% of respondents.

**Fig. 3.**
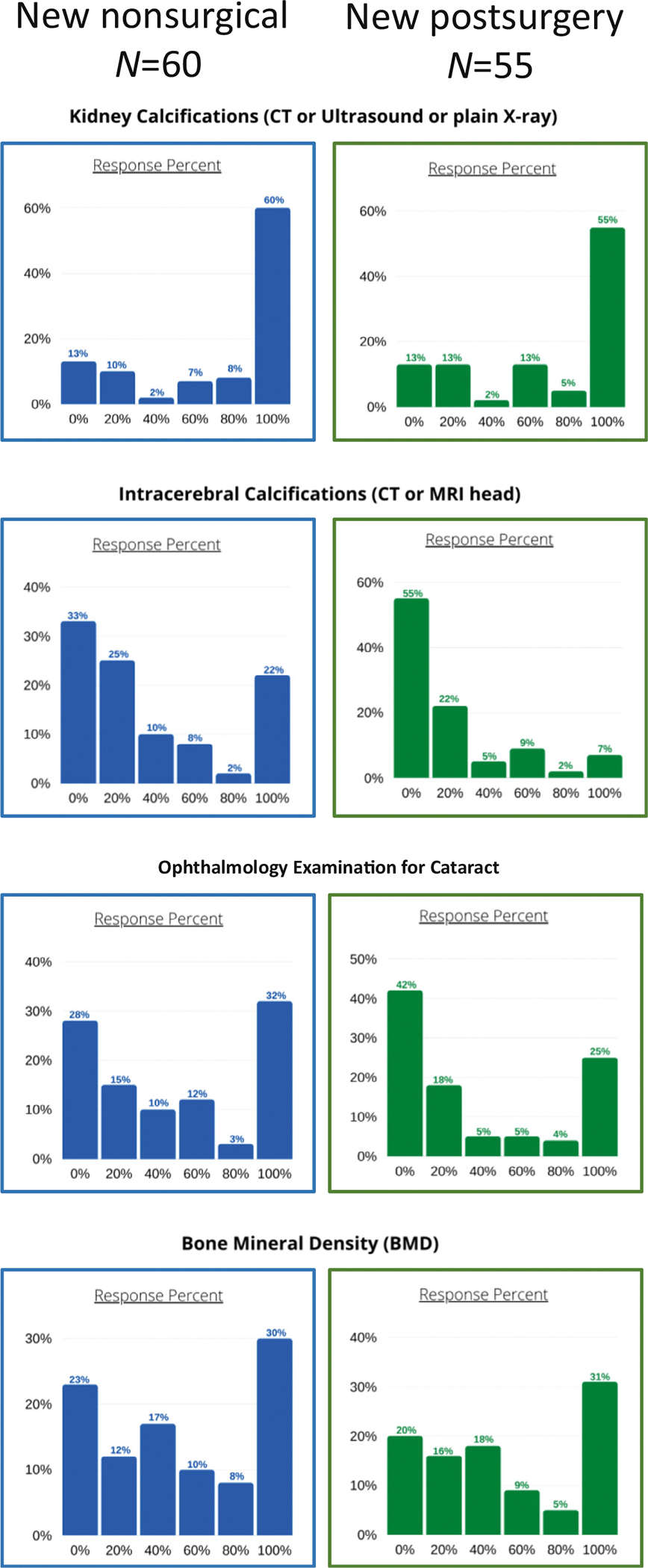
Reported assessment frequency for assessment of chronic complications of HypoPT in new patients. The *x*-axis indicates the percentage of participants on whom assessment of the parameter was performed; the *y*-axis indicates the percentage of respondents who selected this option.

The results of the assessment for potential complications of chronic HypoPT are shown in Figure 3 (left panels). Many respondents perform imaging studies (CT scan, ultrasound, or plain X-ray) to assess for the presence of kidney stones or nephrocalcinosis. Renal imaging studies are completed in all patients by 60% of respondents and in 80% of patients by 8% of respondents. The results of assessment for the presence of other complications, such as intracranial calcifications, cataract, or abnormal BMD, are more heterogeneous.

#### B: Chronic HypoPT postsurgery

Assessment for the presence of anxiety and depression is performed in 100% of patients by 44% of respondents and in 80% of patients by 9% of respondents. The results of biochemical monitoring practice when assessing a new patient with postsurgical chronic HypoPT are shown in Figure 1 (right panels). Similarly to nonsurgical HypoPT, most respondents perform an assessment of serum calcium (either albumin-adjusted calcium or ionized) and albumin. Most participants usually measure serum magnesium, creatinine, phosphate, and 25(OH)D.

The results for 24-hour urine monitoring practice for first assessment of the postsurgical group are shown in Figure 2 (right panels). Similarly to nonsurgical patients, most respondents usually measure 24-hour urinary creatinine and calcium. Measurement of other parameters, including metabolic parameters for kidney stone formation risk, are completed less frequently in most patients. Spot urine test for calcium and creatinine excretion is performed by 22% of respondents.

The results of assessment for potential complications of chronic HypoPT are shown in Figure 3 (right panels). Imaging studies to assess for the presence of nephrocalcinosis (renal CT scan, ultrasound, or plain X-ray) are completed in all patients by 55% of respondents and in 80% of patients by 5% of respondents. The results of assessment for the presence of other complications, including intracranial calcifications, cataract, or abnormal BMD, indicate that many respondents do not perform these assessments on a regular basis.

### Monitoring of follow-up patients

#### Chronic HypoPT, stable

As part of the clinical assessment, most respondents (75%) assess for depression or anxiety every 6 months or less frequently. The responses for biochemical monitoring practice are provided in Table 2. The responses for the postsurgical and nonsurgical groups are very similar. Most respondents perform blood tests on a regular basis, with approximately 60% of respondents completing laboratory profile every 6 months or less frequently, and less than 10% complete a laboratory profile every month or more frequently. With respect to 25(OH)D levels, 81% of respondents measure this parameter every 6 months or less frequently.

The results for 24-hour urine measurements in patients are shown in Table 3. Regarding biochemical parameters, the results are very similar for both postsurgical and nonsurgical patient groups. Most respondents regularly complete a 24-hour urinary measurement for calcium and creatinine clearance (every 6 months or less frequently). The results of monitoring for other urinary metabolic parameters vary considerably among respondents.

The reported clinical practice patterns of monitoring for complications of chronic HypoPT vary considerably among respondents but are rarely completed on a monthly basis (Table 4). There are no major differences between postsurgical and nonsurgical patient groups with regard to monitoring. Standardized QoL questionnaires are utilized by a small number of respondents, with the SF-36 questionnaire being used most frequently (in 9 out of 11 responses).

#### Chronic HypoPT unstable

Many respondents indicated performing blood tests for serum calcium (ionized or albumin-adjusted) and phosphate every month or more frequently in unstable patients, with no major differences between nonsurgical and postsurgical patient groups (Table 2). For all other serum parameters and 24-hour urinary testing and monitoring, there are no major differences in comparison to the assessment pattern for stable patients (Tables 2 and 3, respectively). Similarly, there are no differences in the practice patterns of monitoring for complications of chronic HypoPT (Table 4).

### Review by panel

The evidence-based practice guidance derived from this survey is summarized in Table 5. Our panel reviewed and approved these practices. In addition, the panel decided to add two practice suggestions, which are not survey based. First, the panel suggested performing a baseline assessment with renal imaging for the presence of nephrolithiasis or nephrocalcinosis as this was very close to the 70% criteria and considered clinically relevant for new patients with HypoPT (for all nonsurgical and postsurgical in the presence of risk factors). Factors associated with the rate of chronic kidney disease development include age, duration of disease, proportion of time with relative hypercalcemia, number of hypercalcemic episodes, increased calcium phosphate product, and fractional excretion of phosphate.^(15,16)^ Other renal complications are nephrolithiasis and nephrocalcinosis, and risk factors may include hypercalciuria, elevated calcium phosphate product, and elevated phosphate; however, the association of these risk factors with these complications is not as clearly defined in the HypoPT patient population as it is in the euparathyroid patient population.^(17)^ Secondly, the panel suggests measuring serum calcium (ionized or albumin-adjusted) within days of a change in the medical treatment of chronic HypoPT (as this was felt to be important but not addressed in the survey).

## Discussion

In this survey, we systematically assessed expert practice patterns regarding initial assessment and monitoring of patients with chronic HypoPT. We found several areas of consistency in practice patterns, and other areas with heterogeneity in practice patterns. For the initial assessment, we found consistency with respect to the measurement of several serum parameters, including creatinine, calcium (ionized or albumin-adjusted), magnesium, phosphate, and 25(OH)D, as well as urine parameters (creatinine and calcium). For the monitoring of stable patients with HypoPT, respondents agreed on periodic monitoring of the same parameters. Our survey found no major differences in the initial assessment or follow-up monitoring between postsurgical and nonsurgical HypoPT groups. We reviewed our findings in relation to three published documents on the management of HypoPT in adults, the 2015 European Society for Endocrinology (ESE) Guideline,^(10)^ the 2016 guideline as published by the First International Conference on the Management of HypoPT Statement and Guideline,^(2)^ and the 2019 Canadian and International Consensus Standards on HypoPT.^(3)^ All three groups recommend monitoring serum parameters (calcium [ionized or albumin-adjusted], phosphate, magnesium, and creatinine) with a frequency varying from every 3 to 6 months to annually in stable patients. Our recommendations, which are based on a formal practice survey, are consistent with these previously published recommendations. For patients in whom management is modified, the ESE recommends measuring serum calcium every 1 to 2 weeks, which is consistent with the practice pattern reported in our survey. To ensure patient safety and based on drug pharmacology,^(18)^ we suggest measuring serum calcium within days of making changes in management. This suggestion is made based on consensus as this option (days versus weeks) was not included in the survey. Regarding 24-hour urinary calcium excretion, the ESE suggests assessing this parameter every 1 to 2 years, whereas the two other bodies^(2,3)^ suggest completing this test annually or less frequently when clinically stable. Our practice pattern is consistent with these recommendations.

Major variations are found with respect to the assessment of the development of long-term HypoPT complications. Our survey determined that 60% of participants performed renal imaging in 100% of patients. Most respondents (67% for nonsurgical patients and 76% for postsurgical patients) perform regular renal imaging with a frequency varying from every 6 months to every 2 to 3 years, whereas the present guidelines suggest performing this test based on clinical indications.^(2,10)^ With respect to BMD monitoring, the international conference guidelines^(2)^ suggest monitoring consistent with the International Society of Clinical Densitometry (ISCD) recommendations,^(19)^ whereas the ESE guidelines recommend against routine BMD monitoring.^(10)^ In contrast, the majority of our respondents perform regular BMD studies, with a large number electing to do them every 2 to 3 years.

As indicated by systematic literature reviews that evaluate monitoring recommendations, the currently available evidence for the initial and follow-up monitoring strategies is limited and of low quality. Current guidelines and standards of care clearly acknowledge the limited available evidence for the development of GRADE-based practice recommendations. Therefore, we elected to develop a systematic current practice survey (SCPS) to assess the current practice of participating experts regarding HypoPT monitoring. In our SCPS, we assessed separately the monitoring of patterns at initial evaluation and follow-up of patients with HypoPT. We also evaluated differences in approach for postsurgical versus nonsurgical patients and found no major differences between these groups. We believe that this SCPS reflects both published guideline recommendations and personal experience. Thus, it provides an important assessment of the available evidence and its limitations. In addition, by highlighting significant variations in practice, the SCPS identifies important areas for future research. It should also be recognized that major variations in monitoring for complications of chronic HypoPT may have a significant impact on the reported epidemiology of these complications.

There are several limitations to using the SCPS as a practice assessment tool. Some of these limitations are related to SCPS in general, whereas others are specific to the present study. First, we will address general limitations related to the SCPS. We asked respondents to indicate what their assessment practice was; however, we did not directly evaluate this by performing chart reviews. Therefore, the data are subject to recall bias. We did not assess whether and to what extent practice patterns were based on other factors apart from guidelines and personal knowledge and experience of HypoPT. Monitoring patterns may be influenced by limitations or impositions by the existing health care systems in which the respondents practice. Certain systems may only allow tests to be ordered in “bundles” or impose limitations on test frequency or access to imaging studies.

Second, there are limitations specific to our design of the SCPS. We invited experts (based on HypoPT publications) as well as general endocrinologists without applying any more stringent criteria for participation in the survey. Furthermore, our survey was overrepresented by North American respondents, so not all areas of the world were equally represented. For confidentiality reasons, we were not able to correlate details regarding the respondent practice and their responses (eg, surgeons versus endocrinologists). This limited our ability to determine whether certain response patterns were (sub-) specialty dependent or related to experience or HypoPT practice size. The participants in this survey were composed of endocrinologists (adult and pediatric), nephrologists, and surgeons. We recognize that some of the experts who participated in this survey had a limited number of patients with HypoPT. This could have been due to their focus on research as opposed to clinical practice. A few experts were no longer seeing patients clinically. Panel members were asked to advise regarding their practice pattern in patients who are stable as well as patients who are unstable; however, we did not define clinical stability and left this to the judgment of the participants. We suggest defining clinical stability by (i) an absence of symptoms of hypo- or hypercalcemia or (ii) symptoms that are longstanding, unchanged, and tolerable; (iii) a decreased need for clinic or hospital visits due to stable lab profile (serum and urine calcium, serum phosphorus, and calcium phosphate product) that are in the target range; or (iv) stability in the treatment regimen used. Further, although our analysis is rigorous, it lacks validation. Future studies using the SCPS approach will need to address this. Also, future studies may need to include metrics of bone quality, such as Trabecular Bone Score (TBS) or fracture risk assessment, and assessment for candidacy for second-line treatment, elements that were not included in the current survey. Finally, a recent systematic review^(20)^ found that early postoperative PTH measurements are useful for predicting the risk of chronic HypoPT. This was not assessed in our survey and should be addressed in future SCPS studies.

However, while recognizing the aforementioned limitations, we believe that the SCPS represents a step forward compared to prior consensus and opinion statements. The SCPS provides information on expert practices supported by a systematic research approach. Our survey indicates that the respondents apply similar monitoring strategies for postsurgical and nonsurgical patients with HypoPT. Most respondents had predominantly adults with postsurgical HypoPT in their practice. A recent study demonstrated an increased prevalence of renal complications in nonsurgical versus surgical HypoPT, suggesting that more vigilant monitoring of nonsurgical patients for complications is necessary.^(21)^ In addition, the SCPS identified considerable variation in practice patterns, highlighting areas in need of future research. Our survey recognizes the lack of agreement with respect to the need for initial assessment and follow-up monitoring of complications associated with chronic HypoPT. Another area in need of further study is the indication of measuring urinary metabolic parameters associated with increased risk of kidney stone formation. Finally, a recent study among Dutch/Belgian endocrinologists demonstrated that physicians perceived an impaired QoL in approximately two thirds of patients with HypoPT.^(22)^ However, only a small group of our respondents regularly use QoL questionnaires, and we suggest that more research is needed on the use of HypoPT specific QoL questionnaires.

In conclusion, we developed a SCPS to describe the initial and follow-up assessment of patients with HypoPT by experts in the field. Although serum and urine measurements were made in conformity with published guidelines and consensus statements, the assessment of complications associated with chronic HypoPT varied considerably, and areas for future research were identified. We believe that the SCPS will be helpful in improving the monitoring and management of patients with HypoPT.

## Data Availability Statement

The data that support the findings in this study are openly available in PubMed, MEDLINE, EMBASE, and the Cochrane databases. Detailed survey data is available upon request. Appendixes also included.

## Supplementary Material

appendix 1

Additional Supporting Information may be found in the online version of this article.

## Figures and Tables

**Table 1.** Demographics of Respondents

Demographics of respondents	Total

Practice setting	70
Academic	65 (93%)
Community	5 (7%)
Specialty	70
Pediatric endocrinology	5 (7%)
Adult endocrinology	45 (64%)
Nephrology	2 (3%)
Other	18 (26%)
Do you see…	70
Pediatric patients	
Yes	17 (24%)
No	53 (76%)
Adult patients	
Yes	62 (89%)
No	8 (11%)
Pregnant patients	
Yes	36 (51%)
No	34 (49%)
Years in practice	70
0 to 5 years	4 (6%)
6 to 10 years	5 (7%)
11 to 15 years	6 (8.5%)
16 to 20 years	13 (18.5%)
20 to 25 years	9 (13%)
Over 25 years	33 (47%)
Location	70
North America	36 (51.33%)
South America	4 (6%)
Europe	21 (30%)
Africa	0 (0%)
Asia	8 (11.33%)
Australia and Pacific	1 (1.33%)

**Table 2.** Current Practice for Follow-Up Blood Test Monitoring of Patients with Chronic Hypoparathyroidism

Parameter	Response option	Surgical stable (%)	Nonsurgical stable (%)	Surgical nonstable (%)	Nonsurgical nonstable (%)

Calcium	More often than once monthly	5	2	38	39
	Approximately monthly	5	3	31	34
	Approx. every 3 months	37	37	20	19
	Every 6 months or less frequently	54	58	11	6
Albumin	More often than once monthly	3	2	30	25
	Approximately monthly	2	1	30	31
	Approx. every 3 months	30	27	17	16
	Every 6 months or less frequently	62	65	18	18
Ionized calcium	More often than once monthly	3	2	22	14
	Approximately monthly	2	2	14	21
	Approx. every 3 months	17	13	15	20
	Every 6 months or less frequently	61	63	32	27
Magnesium	More often than once monthly	3	2	22	25
	Approximately monthly	2	3	23	30
	Approx. every 3 months	26	25	27	23
	Every 6 months or less frequently	65	67	26	17
Creatinine	More often than once monthly	3	2	27	26
	Approximately monthly	2	3	19	32
	Approx. every 3 months	33	28	26	22
	Every 6 months or less frequently	62	67	26	15
Phosphate	More often than once monthly	3	2	29	33
	Approximately monthly	3	3	32	38
	Approx. every 3 months	37	38	27	21
	Every 6 months or less frequently	59	55	13	6
25-hydroxyvitamin D	More often than once monthly	2	2	4	5
	Approximately monthly	1	0	4	7
	Approx. every 3 months	12	13	17	17
	Every 6 months or less frequently	81	81	72	64

**Table 3.** Current Practice for Follow-Up 24-Hour Urine Test Monitoring of Patients with Chronic Hypoparathyroidism

					Nonsurgical
Parameter	Response option	Surgical stable (%)	Nonsurgical stable (%)	Surgical nonstable (%)	nonstable (%)

24-hr volume	Never	8	11	12	11
	Every 6 to 12 months	57	55	71	65
	Every 2 years or less frequently	29	27	17	19
	Only if signs/symptoms	6	8	2	2
Calcium	Never	2	2	8	5
	Every 6 to 12 months	72	64	72	72
	Every 2 years or less frequently	22	28	11	19
	Only if signs/symptoms	4	7	4	2
Sodium	Never	21	19	20	17
	Every 6 to 12 months	19	20	24	24
	Every 2 years or less frequently	28	22	22	24
	Only if signs/symptoms	18	26	19	19
Creatinine	Never	10	9	10	9
	Every 6 to 12 months	56	53	67	69
	Every 2 years or less frequently	25	18	14	10
	Only if signs/symptoms	5	13	5	5
Creatinine clearance	Never	13	13	18	12
	Every 6 to 12 months	40	36	43	45
	Every 2 years or less frequently	18	12	12	9
	Only if signs/symptoms	15	25	16	15
Phosphate	Never	21	21	27	21
	Every 6 to 12 months	37	29	37	29
	Every 2 years or less frequently	16	13	10	7
	Only if signs/symptoms	10	24	12	21
Citrate	Never	25	17	23	15
	Every 6 to 12 months	11	6	12	13
	Every 2 years or less frequently	8	16	9	5
	Only if signs/symptoms	40	46	43	48
Oxalate	Never	28	20	30	21
	Every 6 to 12 months	9	5	10	11
	Every 2 years or less frequently	9	14	7	4
	Only if signs/symptoms	41	46	39	45
Uric Acid	Never	23	22	23	20
	Every 6 to 12 months	10	6	10	13
	Every 2 years or less frequently	10	15	11	6
	Only if signs/symptoms	40	40	43	40
Protein	Never	26	24	25	21
	Every 6 to 12 months	11	8	11	17
	Every 2 years or less frequently	11	12	11	5
	Only if signs/symptoms	35	39	37	36

**Table 4.** Current Practice for Monitoring of Patients for Complications of Chronic Hypoparathyroidism

					Nonsurgical
Parameter	Response option	Surgical stable (%)	Nonsurgical stable (%)	Surgical nonstable (%)	Nonstable (%)

Kidney calcifications (CT/ultrasound/X-ray)	Approx. every 6 months	5	4	6	7
	Approx. yearly	25	26	50	34
	Every 2 to 3 years	42	35	19	30
	Only if signs/symptoms	26	33	25	27
Intracerebral calcifications (CT or MRI head)	Approx. every 6 months	2	2	2	2
	Approx. yearly	2	1	3	3
	Every 2 to 3 years	24	23	22	26
	Only if signs/symptoms	64	69	69	64
Ophthalmology evaluation	Approx. every 6 months	2	2	2	2
	Approx. yearly	22	21	24	28
	Every 2 to 3 years	22	19	23	16
	Only if signs/symptoms	48	54	48	47
Bone mineral density	Approx. every 6 months	2	2	2	2
	Approx. yearly	7	6	6	5
	Every 2 to 3 years	55	47	58	53
	Only if signs/symptoms	33	40	32	33
Quality of life questionnaire	Approx. every 6 months	4	4	7	11
	Approx. yearly	3	8	7	6
	Every 2 to 3 years	16	9	7	12
	Only if signs/symptoms	49	47	48	38

**Table 5.** Overview of Practice Suggestions for Patients with Hypoparathyroidism, Both Postsurgery and Nonsurgical

These are graded as low-quality recommendations based on the practice of 70% of the respondents doing this at least 70% of the time. Survey-based practice suggestions for HypoPT Initial assessment • We suggest measurement of serum creatinine, calcium (either ionized or albumin-corrected), magnesium, phosphate, and 25(OH)D • We suggest measurement of 24-hour urine for creatinine (or creatinine clearance) and calcium. Follow-up moitoring Stable patients • We suggest laboratory testing for creatinine, calcium (either ionized calcium or albumin-corrected), phosphate, and magnesium every 3 to 12 months and 25(OH)D every 6 to 12 months. • We suggest 24-hour urine testing for calcium and creatinine (clearance) every 6 months to 2 years. Nonstable patients • We suggest frequent serum calcium and phosphate measurements as clinically indicated. Additional panel recommendations (nonsurvey based). • We suggest baseline assessment for the presence of renal calcifications or stones at first assessment. • We suggest measuring serum calcium (ionized or albumin-corrected) within days of a change in medical treatment of chronic HypoPT.
