# Supplementary material for: Initial Assessment and Monitoring of Patients with Chronic Hypoparathyroidism: A Systematic Current Practice Survey
Source: J Bone Miner Res. Author manuscript; Available in PMC 2026 Jun 4. (PMC13235707; doi:10.1002/jbmr.4698)
Supplement: appendix 1 [file NIHMS2171974-supplement-appendix_1.docx]

**Chronic Hypoparathyroidism Monitoring Survey**

***Version November 28, 2020***

**INTRODUCTION**

Dear Hypoparathyroidism Task Force member,

We invite you to participate in this survey. This is an international initiative with the objective of deriving guidance for monitoring patients with chronic hypoparathyroidism. As you know, currently there are no evidence-based recommendations for monitoring of patients with chronic hypoparathyroidism.

We would like to obtain information on your monitoring practice of patients with chronic hypoparathyroidism and your assessment of frequency of patient outcomes. If you agree to participate, we ask that you choose the responses that are ***closest to your current practice*** (as opposed to recommendations from published guidelines or standards papers).

Completion of the survey will take approximately 1 hour. You will be able to log out and return to continue the survey.

All information will be kept confidential and will only be presented/reported in aggregate format, respecting the confidentiality of individual responses.

Thank you so much for your participation!

Sincerely,

Stan Van Uum

Mohammad Shrayyef

Gordon Guyatt

Aliya Khan and Lars Rejnmark (co-chairs)

**DEMOGRAPHICS OF RESPONDENT**

1. What is your practice setting?
   1. Academic
   2. Community
2. What is your specialty?
   1. pediatric endocrinology
   2. adult endocrinology
   3. nephrology
   4. other
3. Do you see
   1. Pediatric patients with chronic hypoparathyroidism Y/N
   2. Adult patients with chronic hypoparathyroidism Y/N
   3. Pregnant patients with chronic hypoparathyroidism Y/N

*Of note, respondents will only respond to questions about patient groups they see, and not for patient groups they indicate here they do not see.*

1. How many years have you been in practice?
   1. 0-5
   2. 6-10
   3. 11-15
   4. 16-20
   5. 20-25
   6. Over 25
2. How many patients with hypoparathyroidism do you currently have in your practice?
   1. Less than 10
   2. 11 to 25
   3. 26 to 50
   4. 51 to 75
   5. 76 to 100
   6. Over 100
3. Where do you practice?
   1. North-America
   2. South-America
   3. Europe
   4. Africa
   5. Asia
   6. Australia & Pacific

**INTRODUCTION TO CHRONIC HYPOPARATHYROIDISM MONITORING SURVEY**

In the next series of questions, we ask you about your assessment practice for patients with a NEW diagnosis of chronic hypoparathyroidism.

You will do this separately for two groups of patients, non-surgical patients and post-surgical (confirmed with hypoparathyroidism for at least 6 months following surgery) patients with chronic hypoparathyroidism

In addition, we will ask questions about your monitoring practice for pregnant patients – you will be able to opt out if you do not see such patients.

As stated before, we ask that you respond to the question by ***selecting the responses that are closest to your practice.***

You will choose the percentages of new patients in which you perform the assessment indicated. ***NEW PATIENT WITH CHRONIC HYPOPARATHYROIDISM - NON-SURGICAL***

For a NEW patient with chronic non-surgical hypoparathyroidism, how often will you **in your current practice** perform the assessment indicated?

For each parameter **(clinical assessment and blood tests)** listed below, please indicate the proportion of patients in whom you use the strategy indicated?

|  | Proportion |
| --- | --- |
| Clinical assessment | % |
| History for anxiety/depression | % |
| Calcium | % |
| Albumin | % |
| Ionized Calcium | % |
| Magnesium | % |
| Creatinine | % |
| Phosphate | % |
| 25(OH)D | % |

*Percentages from 0 and 100% to be selected using a slider.*

For each **urine test** listed below, please indicate the proportion of patients in whom you use the strategy indicated?

|  | Proportion |
| --- | --- |
| 24-hour volume | % |
| Calcium | % |
| Sodium | % |
| Creatinine | % |
| Creatinine clearance | % |
| Phosphate | % |
| Citrate | % |
| Oxalate | % |
| Uric acid | % |
| Protein | % |

*Percentages from 0 and 100% to be selected using a slider.*

Do you use spot urine testing for screening? **Yes** or **No**

For **each assessment** listed below, please indicate the proportion of patients in whom you use the strategy indicated?

|  | Proportion |
| --- | --- |
| Kidney calcifications (CT or Ultrasound or plain X-ray) | % |
| Intracerebral calcifications (CT or MRI head) | % |
| Cataract or Ophthalmology examination | % |
| Bone Mineral Density (BMD) | % |
| QoL questionnaire* | % |

| % |
| --- |

*If you use a QoL questionnaire, please indicate which one: _________________________________

***NEW PATIENT WITH CHRONIC HYPOPARATHYROIDISM POST SURGERY***

For a NEW patient with chronic hypoparathyroidism post-surgery (at least 6 months post-surgery), how often will you **in your current practice** perform the assessment indicated?

For each parameter **(clinical assessment and blood tests)** listed below, please indicate the proportion of patients in whom you use the strategy indicated?

|  | Proportion |
| --- | --- |
| Clinical assessment | % |
| History for anxiety/depression | % |
| Calcium | % |
| Albumin | % |
| Ionized Calcium | % |
| Magnesium | % |
| Creatinine | % |
| Phosphate | % |
| 25(OH)D | % |

*Percentages from 0 and 100% to be selected using a slider.*

For each **urine test** listed below, please indicate the proportion of patients in whom you use the strategy indicated?

|  | Proportion |
| --- | --- |
| 24-hour volume | % |
| Calcium | % |
| Sodium | % |
| Creatinine | % |
| Creatinine clearance | % |
| Phosphate | % |
| Citrate | % |
| Oxalate | % |
| Uric acid | % |
| Protein | % |

*Percentages from 0 and 100% to be selected using a slider.*

Do you use spot urine testing for screening? **Yes** or **No**

For **each assessment** listed below, please indicate the proportion of patients in whom you use the strategy indicated?

|  | Proportion |
| --- | --- |
| Kidney calcifications (CT or Ultrasound or plain X-ray) | % |
| Intracerebral calcifications (CT or MRI head) | % |
| Cataract or Ophthalmology examination | % |
| Bone Mineral Density (BMD) | % |
| Quality of Life (QoL) questionnaire* | % |

*Percentages from 0 and 100% to be selected using a slider.*

*If you use a QoL questionnaire, please indicate which one: _________________________________

In the next series of questions, we ask you about your monitoring practice for **FOLLOW-UP** of patients with chronic hypoparathyroidism. You will do this separately for four groups of patients. The groups are separated in **non-surgical** and **post-surgical** patients with chronic hypoparathyroidism, and each of these two groups is separated into **‘relatively stable’** and **‘relatively unstable’**, this according to your best clinical judgement.

As stated before, we ask that you respond to the question by ***selecting the responses that are closest to your practice.*** You will choose percentages and create a response distribution, e.g. for monitoring of calcium, you may monitor 20% of your patients every 3 months, 60% every 6 months, and 20% once yearly. Please ensure that the total of these responses adds up to 100%.

***GROUP A***

***CHRONIC HYPOPARATHYROIDISM POST SURGICAL – STABLE***

For a relatively stable follow-up patient with chronic hypoparathyroidism post-surgery (i.e. at least 6 months post-surgery), how often will you **in your current practice** perform the assessment indicated? Note: we will ask you later about unstable patients.

For each parameter **(clinic visit and blood tests)** listed below, please indicate the proportion of patients in whom you use the strategy indicated (percentages in each line need to add up to 100%)?

|  | More often than once monthly | Approximately monthly | Approximately every 3 months | Every 6 months or less frequently | Total  (must be 100%) |
| --- | --- | --- | --- | --- | --- |
| Clinical assessment | % dropdown | % dropdown | % dropdown | % dropdown | SUM |
| History for anxiety/depression | % dropdown | % dropdown | % dropdown | % dropdown | SUM |
| Calcium | % dropdown | % dropdown | % dropdown | % dropdown | SUM |
| Albumin | % dropdown | % dropdown | % dropdown | % dropdown | SUM |
| Ionized Calcium | % dropdown | % dropdown | % dropdown | % dropdown | SUM |
| Magnesium | % dropdown | % dropdown | % dropdown | % dropdown | SUM |
| Creatinine | % dropdown | % dropdown | % dropdown | % dropdown | SUM |
| Phosphate | % dropdown | % dropdown | % dropdown | % dropdown | SUM |
| 25(OH)D | % dropdown | % dropdown | % dropdown | % dropdown | SUM |

*The dropdown list in each cell provided the following options: 0%, 20%, 40%, 60%, 80%, and 100%.*

*The sum of the 4 choices for each parameter must equal 100%.*

For each **urine test** listed below, please indicate the proportion of patients in whom you use the strategy indicated?

|  | Never | Every 6-12 months | Every 2 year or less frequently | Only if signs/symptoms | Total  (must be 100%) |
| --- | --- | --- | --- | --- | --- |
| 24-hour volume | % dropdown | % dropdown | % dropdown | % dropdown | SUM |
| Calcium | % dropdown | % dropdown | % dropdown | % dropdown | SUM |
| Sodium | % dropdown | % dropdown | % dropdown | % dropdown | SUM |
| Creatinine | % dropdown | % dropdown | % dropdown | % dropdown | SUM |
| Creatinine clearance | % dropdown | % dropdown | % dropdown | % dropdown | SUM |
| Phosphate | % dropdown | % dropdown | % dropdown | % dropdown | SUM |
| Citrate | % dropdown | % dropdown | % dropdown | % dropdown | SUM |
| Oxalate | % dropdown | % dropdown | % dropdown | % dropdown | SUM |
| Uric Acid | % dropdown | % dropdown | % dropdown | % dropdown | SUM |
| Protein | % dropdown | % dropdown | % dropdown | % dropdown | SUM |

*The dropdown list in each cell should provide the following options: 0%, 20%, 40%, 60%, 80%, and 100%.*

*The sum of the 4 choices for each parameter must equal 100%.*

Do you use spot urine testing for screening? **Yes** or **No**

For **each assessment** listed below, please indicate the proportion of patients in whom you use the strategy indicated?

|  | Approx. every 6 months | Approx. yearly | Every 2-3 year | Only if signs/ symptoms | Total  (must be 100%) |
| --- | --- | --- | --- | --- | --- |
| Kidney calcifications (CT or ultrasound or plain X-ray) | % dropdown | % dropdown | % dropdown | % dropdown | SUM |
| Intracerebral calcifications (CT or MRI head) | % dropdown | % dropdown | % dropdown | % dropdown | SUM |
| Cataract or Ophthalmology examination | % dropdown | % dropdown | % dropdown | % dropdown | SUM |
| BMD | % dropdown | % dropdown | % dropdown | % dropdown | SUM |
| QoL questionnaire* | % dropdown | % dropdown | % dropdown | % dropdown | SUM |

*The dropdown list in each cell should provide the following options: 0%, 20%, 40%, 60%, 80%, and 100%.*

*The sum of the 4 choices for each parameter must equal 100%.*

*If you use a QoL questionnaire, please indicate which one: _________________________________

***GROUP B***

***CHRONIC HYPOPARATHYROIDISM POST SURGERY – UNSTABLE***

For a relatively unstable follow-up patient with chronic hypoparathyroidism post-surgery, who is on standard of care treatment (calcium supplements and active vitamin D), how often will you **in your current practice** perform monitoring.

For each parameter **(clinic visit and blood tests)** listed below, please indicate the proportion of patients in whom you use the strategy indicated (percentages in each line to add up to 100%)?

|  | More often than once monthly | Approximately monthly | Approximately every 3 months | Every 6 months or less frequently | Total  (must be 100%) |
| --- | --- | --- | --- | --- | --- |
| Clinical assessment | % dropdown | % dropdown | % dropdown | % dropdown | SUM |
| History for anxiety/depression | % dropdown | % dropdown | % dropdown | % dropdown | SUM |
| Calcium | % dropdown | % dropdown | % dropdown | % dropdown | SUM |
| Albumin | % dropdown | % dropdown | % dropdown | % dropdown | SUM |
| Ionized Calcium | % dropdown | % dropdown | % dropdown | % dropdown | SUM |
| Magnesium | % dropdown | % dropdown | % dropdown | % dropdown | SUM |
| Creatinine | % dropdown | % dropdown | % dropdown | % dropdown | SUM |
| Phosphate | % dropdown | % dropdown | % dropdown | % dropdown | SUM |
| 25(OH)D | % dropdown | % dropdown | % dropdown | % dropdown | SUM |

*The dropdown list in each cell should provide the following options: 0%, 20%, 40%, 60%, 80%, and 100%.*

*The sum of the 4 choices for each parameter must equal 100%.*

For each **urine test** listed below, please indicate the proportion of patients in whom you use the strategy indicated?

|  | Never | Every 6-12 months | Every 2 year or less frequently | Only if signs/symptoms | Total  (must be 100%) |
| --- | --- | --- | --- | --- | --- |
| 24-hour volume | % dropdown | % dropdown | % dropdown | % dropdown | SUM |
| Calcium | % dropdown | % dropdown | % dropdown | % dropdown | SUM |
| Sodium | % dropdown | % dropdown | % dropdown | % dropdown | SUM |
| Creatinine | % dropdown | % dropdown | % dropdown | % dropdown | SUM |
| Creatinine clearance | % dropdown | % dropdown | % dropdown | % dropdown | SUM |
| Phosphate | % dropdown | % dropdown | % dropdown | % dropdown | SUM |
| Citrate | % dropdown | % dropdown | % dropdown | % dropdown | SUM |
| Oxalate | % dropdown | % dropdown | % dropdown | % dropdown | SUM |
| Uric Acid | % dropdown | % dropdown | % dropdown | % dropdown | SUM |
| Protein | % dropdown | % dropdown | % dropdown | % dropdown | SUM |

*The dropdown list in each cell should provide the following options: 0%, 20%, 40%, 60%, 80%, and 100%.*

*The sum of the 4 choices for each parameter must equal 100%.*

Do you use spot urine testing for screening? **Yes** or **No**

For **each assessment** listed below, please indicate the proportion of patients in whom you use the strategy indicated?

|  | Approx. every 6 months | Approx. yearly | Every 2-3 year | Only if signs/ symptoms | Total  (must be 100%) |
| --- | --- | --- | --- | --- | --- |
| Kidney calcifications (CT or ultrasound or plain X-ray) | % dropdown | % dropdown | % dropdown | % dropdown | SUM |
| Intracerebral calcifications (CT or MRI head) | % dropdown | % dropdown | % dropdown | % dropdown | SUM |
| Cataract or Ophthalmology examination | % dropdown | % dropdown | % dropdown | % dropdown | SUM |
| BMD | % dropdown | % dropdown | % dropdown | % dropdown | SUM |
| QoL questionnaire* | % dropdown | % dropdown | % dropdown | % dropdown | SUM |

*The dropdown list in each cell should provide the following options: 0%, 20%, 40%, 60%, 80%, and 100%.*

*The sum of the 4 choices for each parameter must equal 100%.*

*If you use a QoL questionnaire, please indicate which one: _________________________________

***GROUP C***

***CHRONIC HYPOPARATHYROIDISM NON-SURGICAL – STABLE***

For a relatively stable follow-up patient with non-surgical chronic hypoparathyroidism, how often will you **in your current practice** perform the assessment indicated?

For each parameter **(clinic visit and blood tests)** listed below, please indicate the proportion of patients in whom you use the strategy indicated (percentages in each line to add up to 100%)?

|  | More often than once monthly | Approximately monthly | Approximately every 3 months | Every 6 months or less frequently | Total  (must be 100%) |
| --- | --- | --- | --- | --- | --- |
| Clinical assessment | % dropdown | % dropdown | % dropdown | % dropdown | SUM |
| History for anxiety/depression | % dropdown | % dropdown | % dropdown | % dropdown | SUM |
| Calcium | % dropdown | % dropdown | % dropdown | % dropdown | SUM |
| Albumin | % dropdown | % dropdown | % dropdown | % dropdown | SUM |
| Ionized Calcium | % dropdown | % dropdown | % dropdown | % dropdown | SUM |
| Magnesium | % dropdown | % dropdown | % dropdown | % dropdown | SUM |
| Creatinine | % dropdown | % dropdown | % dropdown | % dropdown | SUM |
| Phosphate | % dropdown | % dropdown | % dropdown | % dropdown | SUM |
| 25(OH)D | % dropdown | % dropdown | % dropdown | % dropdown | SUM |

*The dropdown list in each cell should provide the following options: 0%, 20%, 40%, 60%, 80%, and 100%.*

*The sum of the 4 choices for each parameter must equal 100%.*

For each **urine test** listed below, please indicate the proportion of patients in whom you use the strategy indicated?

|  | Never | Every 6-12 months | Every 2 year or less frequently | Only if signs/symptoms | Total  (must be 100%) |
| --- | --- | --- | --- | --- | --- |
| 24-hour volume | % dropdown | % dropdown | % dropdown | % dropdown | SUM |
| Calcium | % dropdown | % dropdown | % dropdown | % dropdown | SUM |
| Sodium | % dropdown | % dropdown | % dropdown | % dropdown | SUM |
| Creatinine | % dropdown | % dropdown | % dropdown | % dropdown | SUM |
| Creatinine clearance | % dropdown | % dropdown | % dropdown | % dropdown | SUM |
| Phosphate | % dropdown | % dropdown | % dropdown | % dropdown | SUM |
| Citrate | % dropdown | % dropdown | % dropdown | % dropdown | SUM |
| Oxalate | % dropdown | % dropdown | % dropdown | % dropdown | SUM |
| Uric Acid | % dropdown | % dropdown | % dropdown | % dropdown | SUM |
| Protein | % dropdown | % dropdown | % dropdown | % dropdown | SUM |

*The dropdown list in each cell should provide the following options: 0%, 20%, 40%, 60%, 80%, and 100%.*

*The sum of the 4 choices for each parameter must equal 100%.*

Do you use spot urine testing for screening? **Yes** or **No**

For **each assessment** listed below, please indicate the proportion of patients in whom you use the strategy indicated?

|  | Approx. every 6 months | Approx. yearly | Every 2-3 year | Only if signs/ symptoms | Total  (must be 100%) |
| --- | --- | --- | --- | --- | --- |
| Kidney calcifications (CT or ultrasound or plain X-ray) | % dropdown | % dropdown | % dropdown | % dropdown | SUM |
| Intracerebral calcifications (CT or MRI head) | % dropdown | % dropdown | % dropdown | % dropdown | SUM |
| Cataract or Ophthalmology examination | % dropdown | % dropdown | % dropdown | % dropdown | SUM |
| BMD | % dropdown | % dropdown | % dropdown | % dropdown | SUM |
| QoL questionnaire* | % dropdown | % dropdown | % dropdown | % dropdown | SUM |

*The dropdown list in each cell should provide the following options: 0%, 20%, 40%, 60%, 80%, and 100%.*

*The sum of the 4 choices for each parameter must equal 100%.*

*If you use a QoL questionnaire, please indicate which one: _________________________________

***GROUP D***

***CHRONIC HYPOPARATHYROIDISM NON-SURGICAL – UNSTABLE***

For a relatively unstable follow-up patient with non-surgical chronic hypoparathyroidism, who is on standard of care treatment (calcium supplements and active vitamin D), how often will you **in your current practice** perform monitoring.

For each parameter **(clinic visit and blood tests)** listed below, please indicate the proportion of patients in whom you use the strategy indicated (percentages in each line to add up to 100%)?

|  | More often than once monthly | Approximately monthly | Approximately every 3 months | Every 6 months or less frequently | Total  (must be 100%) |
| --- | --- | --- | --- | --- | --- |
| Clinical assessment | % dropdown | % dropdown | % dropdown | % dropdown | SUM |
| History for anxiety/depression | % dropdown | % dropdown | % dropdown | % dropdown | SUM |
| Calcium | % dropdown | % dropdown | % dropdown | % dropdown | SUM |
| Albumin | % dropdown | % dropdown | % dropdown | % dropdown | SUM |
| Ionized Calcium | % dropdown | % dropdown | % dropdown | % dropdown | SUM |
| Magnesium | % dropdown | % dropdown | % dropdown | % dropdown | SUM |
| Creatinine | % dropdown | % dropdown | % dropdown | % dropdown | SUM |
| Phosphate | % dropdown | % dropdown | % dropdown | % dropdown | SUM |
| 25(OH)D | % dropdown | % dropdown | % dropdown | % dropdown | SUM |

*The dropdown list in each cell should provide the following options: 0%, 20%, 40%, 60%, 80%, and 100%.*

*The sum of the 4 choices for each parameter must equal 100%.*

For each **urine test** listed below, please indicate the proportion of patients in whom you use the strategy indicated?

|  | Never | Every 6-12 months | Every 2 year or less frequently | Only if signs/symptoms | Total  (must be 100%) |
| --- | --- | --- | --- | --- | --- |
| 24-hour volume | % dropdown | % dropdown | % dropdown | % dropdown | SUM |
| Calcium | % dropdown | % dropdown | % dropdown | % dropdown | SUM |
| Sodium | % dropdown | % dropdown | % dropdown | % dropdown | SUM |
| Creatinine | % dropdown | % dropdown | % dropdown | % dropdown | SUM |
| Creatinine clearance | % dropdown | % dropdown | % dropdown | % dropdown | SUM |
| Phosphate | % dropdown | % dropdown | % dropdown | % dropdown | SUM |
| Citrate | % dropdown | % dropdown | % dropdown | % dropdown | SUM |
| Oxalate | % dropdown | % dropdown | % dropdown | % dropdown | SUM |
| Uric Acid | % dropdown | % dropdown | % dropdown | % dropdown | SUM |
| Protein | % dropdown | % dropdown | % dropdown | % dropdown | SUM |

*The dropdown list in each cell should provide the following options: 0%, 20%, 40%, 60%, 80%, and 100%.*

*The sum of the 4 choices for each parameter must equal 100%.*

Do you use spot urine testing for screening? **Yes** or **No**

For **each assessment** listed below, please indicate the proportion of patients in whom you use the strategy indicated?

|  | Approx. every 6 months | Approx. yearly | Every 2-3 year | Only if signs/ symptoms | Total  (must be 100%) |
| --- | --- | --- | --- | --- | --- |
| Kidney calcifications (CT or ultrasound or plain X-ray) | % dropdown | % dropdown | % dropdown | % dropdown | SUM |
| Intracerebral calcifications (CT or MRI head) | % dropdown | % dropdown | % dropdown | % dropdown | SUM |
| Cataract or Ophthalmology examination | % dropdown | % dropdown | % dropdown | % dropdown | SUM |
| BMD | % dropdown | % dropdown | % dropdown | % dropdown | SUM |
| QoL questionnaire* | % dropdown | % dropdown | % dropdown | % dropdown | SUM |

*The dropdown list in each cell should provide the following options: 0%, 20%, 40%, 60%, 80%, and 100%.*

*The sum of the 4 choices for each parameter must equal 100%.*

*If you use a QoL questionnaire, please indicate which one: _________________________________

***GROUP E***

***CHRONIC HYPOPARATHYROIDISM PREGNANT***

If you do not see hypoparathyroid patients who become pregnant, please click the next button to opt out

(radiobutton) I do not see these patients ( -> will skip this section)

For a pregnant patient with chronic hypoparathyroidism, who is on standard of care treatment (calcium supplements and active vitamin D), how often will you **in your current practice** perform monitoring.

For each parameter **(clinic visit and** **blood tests)** listed below, please indicate the proportion of patients in whom do you use the strategy indicated?

|  | More often than once monthly | Approximately monthly | Approximately every 3 months | Every 6 months or less frequently | Total  (must be 100%) |
| --- | --- | --- | --- | --- | --- |
| Clinical assessment | % dropdown | % dropdown | % dropdown | % dropdown | SUM |
| History for anxiety/depression | % dropdown | % dropdown | % dropdown | % dropdown | SUM |
| Calcium | % dropdown | % dropdown | % dropdown | % dropdown | SUM |
| Albumin | % dropdown | % dropdown | % dropdown | % dropdown | SUM |
| Ionized Calcium | % dropdown | % dropdown | % dropdown | % dropdown | SUM |
| Magnesium | % dropdown | % dropdown | % dropdown | % dropdown | SUM |
| Creatinine | % dropdown | % dropdown | % dropdown | % dropdown | SUM |
| Phosphate | % dropdown | % dropdown | % dropdown | % dropdown | SUM |
| 25(OH)D | % dropdown | % dropdown | % dropdown | % dropdown | SUM |

*The dropdown list in each cell should provide the following options: 0%, 20%, 40%, 60%, 80%, and 100%.*

*The sum of the 4 choices for each parameter must equal 100%.*

For each **urine test** listed below, please indicate the proportion of patients in whom you use the strategy indicated?

|  | Never | Every 6-12 months | Every 2 year or less frequently | Only if signs/symptoms | Total  (must be 100%) |
| --- | --- | --- | --- | --- | --- |
| 24-hour volume | % dropdown | % dropdown | % dropdown | % dropdown | SUM |
| Calcium | % dropdown | % dropdown | % dropdown | % dropdown | SUM |
| Sodium | % dropdown | % dropdown | % dropdown | % dropdown | SUM |
| Creatinine | % dropdown | % dropdown | % dropdown | % dropdown | SUM |
| Creatinine clearance | % dropdown | % dropdown | % dropdown | % dropdown | SUM |
| Phosphate | % dropdown | % dropdown | % dropdown | % dropdown | SUM |
| Citrate | % dropdown | % dropdown | % dropdown | % dropdown | SUM |
| Oxalate | % dropdown | % dropdown | % dropdown | % dropdown | SUM |
| Uric Acid | % dropdown | % dropdown | % dropdown | % dropdown | SUM |
| Protein | % dropdown | % dropdown | % dropdown | % dropdown | SUM |

*The dropdown list in each cell should provide the following options: 0%, 20%, 40%, 60%, 80%, and 100%.*

*The sum of the 4 choices for each parameter must equal 100%.*

Do you use spot urine testing for screening? **Yes** or **No**

For **each assessment** listed below, please indicate the proportion of patients in whom you use the strategy indicated?

|  | Never | Approx. every 3-6 months | Only if signs/ symptoms | Total  (must be 100%) |
| --- | --- | --- | --- | --- |
| Kidney calcifications (CT or ultrasound or plain X-ray) | % dropdown | % dropdown | % dropdown | SUM |
| Intracerebral calcifications (CT head) | % dropdown | % dropdown | % dropdown | SUM |
| Cataract / Ophthalmology examination | % dropdown | % dropdown | % dropdown | SUM |
| QoL questionnaire* | % dropdown | % dropdown | % dropdown | SUM |

*The dropdown list in each cell should provide the following options: 0%, 20%, 40%, 60%, 80%, and 100%.*

*The sum of the 3 choices for each parameter must equal 100%.*

*If you use a QoL questionnaire, please indicate which one: _________________________________

In the next set of questions, we ask that you indicate your experience on the occurrence of outcomes in your patients with hypoparathyroidism.

**OUTCOMES HYPOPARATHYROIDISM – SURGICAL**

Please consider all patients with surgical hypoparathyroidism you have followed in the last decade.

What proportion of these patients have, during follow-up, had (at least once) had an ER visit or hospital admission for hypocalcaemia?

- Less than 5%
- 6 - 10%
- 11 - 25%
- Over 25%

(radiobuttons)

What proportion of these patients have, during follow-up, had at least one major seizure?

- Less than 1%
- 1-5%
- 6-15%
- Over 15%

(radiobuttons)

What proportion of these patients have, during follow-up, developed kidney stones?

- Less than 1%
- 1 to 5%
- 6-15%
- Over 15%

(radiobuttons)

What proportion of these patients have, during follow-up, developed nephrocalcinosis?

- Less than 1%
- 1 to 5%
- 6-15%
- Over 15%

(radiobuttons)

What proportion of these patients have, during follow-up, developed chronic renal failure required dialysis or kidney transplant?

- Less than 1%
- 1 to 5%
- 6-15%
- Over 15%

(radiobuttons)

What proportion of these patients have, during follow-up, developed an osteoporotic fracture?

- Less than 1%
- 1 to 5%
- 5-15%
- Over 15%

(radiobuttons)

What proportion of these patients have, during follow-up, developed cataract requiring surgery?

- Less than 1%
- 1 to 5%
- 6-15%
- Over 15%

(radiobuttons)

**OUTCOMES HYPOPARATHYROIDISM – NON-SURGICAL**Please consider all patients with non-surgical hypoparathyroidism you have followed in the last decade.

What proportion of these patients have, during follow-up, had (at least once) had an ER visit or hospital admission for hypocalcaemia?

- Less than 5%
- 5 - 10%
- 11 - 25%
- Over 25%

(radiobuttons)

What proportion of these patients have, during follow-up, had at least one major seizure?

- Less than 1%
- 1-5%
- 6-15%
- Over 15%

(radiobuttons)

What proportion of these patients have, during follow-up, developed kidney stones?

- Less than 1%
- 1 to 5%
- 6-15%
- Over 15%

(radiobuttons)

What proportion of these patients have, during follow-up, developed nephrocalcinosis?

- Less than 1%
- 1 to 5%
- 6-15%
- Over 15%

(radiobuttons)

What proportion of these patients have, during follow-up, developed chronic renal failure required dialysis or kidney transplant?

- Less than 1%
- 1 to 5%
- 6-15%
- Over 15%

(radiobuttons)

What proportion of these patients have, during follow-up, developed an osteoporotic fracture?

- Less than 1%
- 1 to 5%
- 6-15%
- Over 15%

(radiobuttons)

What proportion of these patients have, during follow-up, developed cataract requiring surgery?

- Less than 1%
- 1 to 5%
- 6-15%
- Over 15%

(radiobuttons)

**OUTCOMES HYPOPARATHYROIDISM – PREGNANCY**

If you do not see hypoparathyroid patients who become pregnant, please click the next button to opt out

(radiobutton) I do not see these patients ( -> will skip this section)

Please consider all pregnant patients with hypoparathyroidism who you have followed in the last decade.

What proportion of these patients have, during follow-up, had (at least once) had an ER visit or hospital admission for hypocalcaemia?

- Less than 5%
- 6 - 10%
- 11 - 25%
- Over 25%

(radiobuttons)

What proportion of these patients have, during follow-up, had at least one major seizure?

- Less than 1%
- 1-5%
- 6-15%
- Over 15%

(radiobuttons)

What proportion of these patients have, during follow-up, had abortion in pregnancy?

- Less than 1%
- 1 to 5%
- 6-15%
- Over 15%

(radiobuttons)

What proportion of these patients have, during follow-up, experience preterm labours?

- Less than 1%
- 1 to 5%
- 6-15%
- Over 15%

(radiobuttons)

What proportion of these patients have, after delivery, reported hypercalcemia in the neonate?

- Less than 1%
- 1 to 5%
- 6-15%
- Over 15%

(radiobuttons)

What proportion of these patients have, after delivery, reported hypocalcemia in the neonate?

- Less than 1%
- 1 to 5%
- 6-15%
- Over 15%

(radiobuttons)
